# Maternal Sleep Disturbances as an Upstream Modulator of the Immunological Biomarker Profile in Early-Onset Preeclampsia

**DOI:** 10.34763/jmotherandchild.20263001.d-26-00016

**Published:** 2026-09-25

**Authors:** Roheen Raza

**Affiliations:** Dow University of Health Sciences, Karachi, Pakistan

Dear Editor,

The recently published article by Dash et al. (2026) makes a valuable contribution to early preeclampsia prediction by demonstrating that combined CD4^+^ FOXP3^+^ T-regulatory cell count and IL-6 achieves an area under the curve of 0.994, establishing the diagnostic primacy of immune-inflammatory dysregulation in early-onset disease.

Despite this precision, one clinically significant upstream exposure remains unexamined: maternal sleep health. Sleep disturbances affect up to 80% of pregnant women yet are absent from standard preeclampsia risk stratification frameworks. This omission is consequential because the immunological pathways central to Dash et al.’s panel — IL-6, hsCRP, and CD4^+^ FOXP3^+^ T-regulatory cells are demonstrably perturbed by chronic sleep disruption through at least three converging mechanisms. First, sleep deprivation elevates circulating IL-6 and CRP via hypothalamic-pituitary-adrenal axis activation and sympathetic nervous system upregulation [1]. Second, obstructive sleep apnoea is present in up to 26% of pregnant women, which independently suppresses CD4^+^ FOXP3^+^ T-regulatory cell populations through intermittent hypoxia-driven oxidative stress and NF-κB-mediated inflammation [2]. Third, circadian disruption reduces melatonin bioavailability, impairing its antioxidant and immunomodulatory protection of trophoblast function and spiral artery remodelling [3]. Maternal sleep disturbances may therefore prime the same immunological milieu that Dash et al. identify as central to early-onset preeclampsia pathogenesis.

The study’s additional limitations are that it is a single-centre design, a small sample of 40 participants, and a cross-sectional methodology precluding temporal inference that further underscore the need for prospective multicentre cohort studies [4]. Such studies should incorporate objective sleep assessment using actigraphy or polysomnography from the first trimester, alongside serial measurement of the T-reg/IL-6/hsCRP biomarker panel. This design would establish whether sleep-related immune dysregulation constitutes an independent, modifiable upstream contributor to the preeclamptic phenotype described by Dash et al., and whether targeted sleep interventions could shift these biomarker trajectories before clinical disease emerges.

Identifying maternal sleep disturbance as a modifiable antecedent of immune dysregulation could open a tractable and low-cost prevention window in preeclampsia management.
